# Use of misoprostol in the treatment of postpartum hemorrhage: a pharmacoepidemiological approach

**DOI:** 10.31744/einstein_journal/2020AO5029

**Published:** 2019-10-28

**Authors:** Daeska Marcella Koch, Yanna Dantas Rattmann

**Affiliations:** 1 Universidade Federal do Paraná, Curitiba, PR, Brazil.

**Keywords:** Misoprostol, Delivery, obstetric, Pharmacoepidemiology, Postpartum hemorrhage, Treatment outcome

## Abstract

**Objective:**

To characterize the use of the drug misoprostol for treatment of postpartum hemorrhage in pregnant women.

**Methods:**

A descriptive observational study was carried out with secondary data from pregnant women who used misoprostol to treat postpartum hemorrhage in a reference public maternity, from July 2015 to June 2017. Clinical and sociodemographic profiles of pregnant women, how misoprostol was used and success rate in controling postpartum hemorrhage were characterized.

**Results:**

A total of 717 prescriptions of misoprostol were identified. Of these, 10% were for treatment of postpartum hemorrhage. The majority of pregnant women were young adults, married, with complete high school education, white, residing in urban areas, multiparous (68.1%) and 25% had previous cesarean sections. The mean gestational age was 39 weeks and 51.4% had a cesarean section. There was prophylactic use of oxytocin in 47.2% of women. Treatment of postpartum hemorrhage was successful in 84.7% of women. Of these, 79.2% also used oxytocin and 54.2% methylergonovine. Only 13.5% of pregnant women had less than five prenatal visits, and the main cause of postpartum hemorrhage was uterine atony. There were 13 complications after hemorrhage, 15.3% required blood transfusion and there was one case of maternal death.

**Conclusion:**

Misoprostol showed to be effective and safe for treating postpartum hemorrhage.

## INTRODUCTION

According to the World Health Organization (WHO), postpartum hemorrhage (PPH) is the cause of approximately 25% of all deaths of pregnant women all over the world, especially in low-income countries. It is also responsible for the majority of severe maternal morbidities, such as prolonged hospital admissions, need for blood transfusions and surgical procedures that can lead to loss of reproductive function.^1^ In Brazil, bleeding is the second leading cause of maternal death, and PPH accounts for 40.8% of total number of obstetric hemorrhages.^2^

Most of those deaths can be avoided by prophylactic use of oxytocic agents during the third stage of delivery and rapid and appropriate management of bleeding. Nevertheless, on average, 6% of all deliveries progress to PPH, and 1.86% to severe PPH (≥1,000mL blood loss).^3^

The major cause of PPH is uterine atony (80% of cases). Lacerations of the birth canal or perineum, uterine inversion, maternal coagulation disorders, placental retention, are some other causes.^4^ If PPH occurs consequent to uterine atony, the initial treatment consists of uterine massage, followed by use of oxytocic agents, such as oxytocin, ergometrine, prostaglandins, and analogs.^2^

Among the oxytocic drugs, the first choice for treatment of PPH is intravenous oxytocin. If it is unavailable or bleeding persists, ergometrine or a fixed dose of the combination oxytocin-ergometrine is recommended. As third option, prostaglandin or its analogs, such as misoprostol, is used.^2 , 5^

Due to its uterotonic effect, misoprostol has been investigated as an alternative to oxytocin, since it is easy to administer, stable at room temperature, and affordable. In 2006, the International Federation of Gynecology and Obstetrics (FIGO) recommended the use of misoprostol to treat PPH, especially in locations with scarce resources.^6^ In 2012, FIGO published its guidelines indicating 800µg misoprostol for the treatment of PPH.^7^ However, it was not until 2015 that the WHO included this drug on the list of essential drugs for this purpose.^8^

Misoprostol is a prostaglandin E1 analogue originally developed for the treatment of peptic and duodenal ulcers. In the 1990´s, Cytotec^®^, its commercial name at the time, could be acquired in drugstores or illegal commerce, becoming popular in Brazil as a widely used method to induce abortion. As from 1998, the drug has been subject to special control in the country, and its use is restricted to hospitals licensed by the health authority.^9 , 10^ It is currently considered by the Brazilian Federation of Gynecology and Obstetrics Associations (Febrasgo)^10^ and by the Ministry of Health,^11^ as a pharmacological alternative for cervical maturation and induction of delivery with live fetus; for termination of pregnancy of anencephalic fetus; termination when there is maternal risk or pregnancy resulting from rape;^12^ for retained and incomplete abortions; and for the treatment of PPH.

Following administration (oral, sublingual, vaginal or rectal), misoprostol undergoes de-esterification in the liver, changing into misoprostol acid. This active metabolite acts directly on prostaglandin receptors, causing changes in the physical-chemical structure of the cervix collagen, which leads to softening and maturation of the cervix, favoring its dilation. It also promotes increased intracellular calcium, responsible for contraction of the myometrial muscle. The most common adverse reactions associated with misoprostol are nausea, vomiting, diarrhea, abdominal pain, fever and chills. Severe reactions are rare and include tachysystole, hypercontractility, and uterine rupture. All of these reactions are dose-dependent and tend to decrease in intensity in the first hours after use.^10 - 13^

## OBJECTIVE

To identify the clinical and epidemiological profiles of the use of misoprostol for treatment of postpartum hemorrhage.

## METHODS

A cross-sectional descriptive observational study aimed to study the pattern of use and success rate of misoprostol for treating PPH in all pregnant women who used this drug at a maternity hospital in the State of Paraná, Brazil. This hospital is part of the Brazilian National Health System (SUS - *Sistema Único de Saúde* )and the obstetric department is the reference center for high-risk pregnancy for the city and the metropolitan region. Approximately 3,000 births occur each year at the hospital.

The study period was from July 2015, when the Health Surveillance agency authorized the use of misoprostol in the organization, to June 2017. The clinical profile of the neonates of these pregnant women was also characterized, for the possibility of a correlation with PPH.

The analysis of success rate (effectiveness) of misoprostol considered cessation of bleeding, with no need for further complementary methods, or no occurrence of complications, such as hysterectomy after the use of the drug.

The WHO,^1^ FIGO^7^ and the Ministry of Health^11^ recommended up to 800µg of misoprostol for treatment of PPH, given as up to four consecutive 200µg tablets. Although misoprostol tablets were developed for vaginal use, the preferred routes of administration for PPH are sublingual or rectal. The hospital adopted the rectal route as routine.

Data were collected using the misoprostol order form, which provided information on maternal age, gestational age (calculated from ultrasound, in full weeks), justification for use (through the International Statistical Classification of Diseases and Related Health Problems - ICD-10), and dosage; medical prescriptions with dosage, duration of treatment and use of other oxytocic drugs (methylergomevine and/or oxytocin); the computerized system (color/race, education, marital status and place of residence); and the electronic medical record, containing the type of delivery, justification for cesarean section, success with the use of the drug, previous pregnancies, parity, complications in delivery or postpartum period, conditions during pregnancy (maternal comorbidities and conditions in the fetus), number of prenatal visits, and other auxiliary methods used for delivery. Data of the neonate (Apgar in the first and fifth minutes of life, and birth weight) were also collected for analysis.

The collected data were input in Microsoft Excel^®^, with further descriptive analysis by Epi Info application (version 6.04).

The study was approved by the Internal Review Board of the *Universidade Federal do Paraná* , Divison of Health Sciences, according to the opinion 017569/2017, CAAE: 65363617.2.0000.0102.

## RESULTS

Misoprostol was given to 717 pregnant women, of whom 362 received the medication to induce delivery with live fetus (50.5%), 283 to manage abortion and pregnancy termination with retained dead fetus (39.5%), and 72 to treat PPH (10.0%).

Sociodemographic characteristics and clinical information of the pregnant women who used misoprostol for treatment of PPH are depicted in tables 1 and 2 , respectively.

**Table 1:** Sociodemographic variables of the pregnant women treated with misoprostol for postpartum hemorrhage

Variables	
Age group (years)	
<20	10 (13.9)
20-34	45 (62.5)
>34	17 (23.6)
Marital status	
Single	15 (20.8)
Married/ *de facto* relationship	47 (65.3)
Widow/divorced	0 (0)
Not informed	10 (13.9)
Schooling	
Literate	1 (1.4)
Incomplete Elementary School	9 (12.5)
Complete Elementary School	10 (13.9)
Incomplete High School	5 (6.9)
Complete High School	12 (16.7)
Incomplete Higher Education	5 (6.9)
Complete Higher Education	1 (1.4)
Not informed	29 (40.3)
Race/color	
White	37 (51.4)
Brown	31 (43.0)
Black	1 (1.4)
Yellow	1 (1.4)
Not informed	2 (2.8)
Area of residence	
Urban	52 (72.2)
Rural	20 (27.8)

Results expressed as n (%).

**Table 2:** Clinical variables of the pregnant women treated with misoprostol for postpartum hemorrhage

Variables	
Gestational age, weeks	
<12	1 (1.4)
12-36	10 (13.9)
37-41	61 (84.7)
Parity	
0	23 (31.9)
1	23 (31.9)
2	11 (15.3)
3	5 (6.9)
4	5 (6.9)
5	3 (4.2)
6	2 (2.8)
Multiple pregnancy	3 (4.2)
History of vaginal delivery	32 (44.4)
History of abortion	16 (22.2)
History of C-section	18 (25.0)

Results expressed as n (%).

The mean age was 28.51±7.46 years, range 16 to 45 years; median gestational age was 39 weeks, range of 7 to 41 weeks.

The success rate of misoprostol for treatment of PPH, associated or not with other drugs, was 84.7%; it was used as the third option in 37 cases (51.4%), as a second option in 22 cases (30.5%), and as single drug in 13 cases (18.0%). Misoprostol as monotherapy failed in only one pregnant woman, requiring surgical intervention and hysterectomy ( Table 3 ).

**Table 3:** Variables related to delivery and use of medications by pregnant women treated with misoprostol for postpartum hemorrhage

Variables	
Delivery	
Vaginal	34 (47.2)
Cesarean section	37 (51.4)
Others (curettage)	1 (1.4)
Prophylactic use of oxytocin	34 (47.2)
Use of oxytocin after PPH	57 (79.2)
Use of methylergometrine after PPH	39 (54.2)
Number of 200µg misoprostol pills	
1	3 (4.2)
2	10 (13.9)
3	1 (1.4)
4	56 (77.8)
5	1 (1.4)
6	1 (1.4)
Success rate after misoprostol use	61 (84.7)

Results expressed as n (%). PPH: postpartum hemorrhage.

Out of 11 cases of PPH that failed, all used oxytocin as the first option, five used methylergomevine, and all used misoprostol, ranging the dosage as one (n=1), two (n=1), three (n=1) and four 200µg tablets (n=8).

Of the women who used methylergometrine (54%), four had hypertension, which is a contraindication for this medication.

According to the medical records, six cases of PPH were due to laceration of the birth canal (8.3%), one case due to perforation of the uterine wall (during curettage; 1.4%), two cases were secondary PPH (which occurs between 24 hours and 6 weeks after delivery due to placental remains or uterine infection; 2.8%), three were due to coagulation disorder (4.2%), and the remaining (60 cases; 83.3%) to uterine atony. A total of 11 (15.3%) pregnant women required blood transfusion after PPH.

Of the 72 pregnant women treated for PPH, 51.4% had cesarean section, mostly due to cephalopelvic disproportion (13.5%), failure to induce vaginal delivery (13.5%), and fetal macrosomia (8.1%).

Twenty-four different ICD codes were reported for the use of the drug. Among them, approximately 28% corresponded to O72.1 (other immediate postpartum hemorrhages), 15.3% to O72 (PPH) and 9.7% to O80.0 (full-term uncomplicated delivery).

Regarding the number of prenatal visits, 48.6% of women had between six and ten visits, 22.2% more than ten, 15.3% had no data registered, and 13.9% had two to five consultations.

As to neonate data, there were 5.3% fetal deaths, 12.0% cases of macrosomia (weight ≥4,000 g), and 8.0% with low weight (<2,500 g). Of the total, 82.7% were born with 1-minute Apgar score above, and 89.3% with 5-minute Apgar score above 7.

Thirteen maternal complications and hemorrhage treatment after partum were identified, including hysterectomy (n=4), hypovolemic shock with subsequent admission to the intensive care unit (n=4), anemia (n=2), postpartum infection (n=2), and maternal death (n=1).

Among the pre-existing and gestational conditions, 121 cases were found in 72 pregnant women, as shown in table 4 . The most prevalent were pregnancy-induced hypertension (PIH) (12.4%), followed by hypothyroidism and obesity, 9.1% each.

**Table 4:** Preexisting and gestational conditions of pregnant women treated with misoprostol for postpartum hemorrhage (n=121)

Existing conditions	
Pregnancy-induced hypertension	15 (12.4)
Hypothyroidism	11 (9.1)
Obesity	11 (9.1)
Urinary tract infection	10 (8.3)
Premature delivery	10 (8.3)
Hypertension	7 (5.8)
Vaginal infection	7 (5.8)
Neurologic disorders*	6 (5.0)
Smoking	5 (4.1)
Edema	4 (3.3)
*Diabetes mellitus*	3 (2.5)
Respiratory tract disease	3 (2.5)
Multiple pregnancy	3 (2.5)
Bowel infection	3 (2.5)
Thrombocytopenia	3 (2.5)
Pre-eclampsia	3 (2.5)
Anemia	2 (1.7)
Amniotic fluid disorder	2 (1.7)
Syphilis	2 (1.7)
Uterine varices	2 (1.7)
Bartholinitis	1 (0.8)
Chorioamnionitis	1 (0.8)
Placental abruption	1 (0.8)
Former smoker	1 (0.8)
Eclampsia	1 (0.8)
Umbilical hernia	1 (0.8)
Hyperemesis gravidarum	1 (0.8)
Vaginal bleeding	1 (0.8)
Bicornuate uterus	1 (0.8)
Total	121 (100.0)

Results expressed as n (%). * Panic syndrome, depression, anxiety, schizophrenia, and/or epilepsy.

In this study, all pregnant women had at least one risk factor that could result in in PPH, such as age under 20 or above 35 years (37.5%); multiparity (68.1%), multiple pregnancy (4.2%), induced delivery (1.4%), amniotic fluid disorders (2.8%), fetal macrosomia (12%), premature labor (13, 9%), gestational anemia or thrombocytopenia (6.9%), hypertensive disorders (36.1%), chorioamnionitis (1.4%), placental abruption (1.4%), obesity (15.3%), delivery associated with uterine infection (1.4%), and dystocia (2.8%). Vaginal delivery was found in 47.2% of pregnant women.

## DISCUSSION

Hemorrhage is one of the leading preventable causes of maternal death.^1^ When PPH is diagnosed, in parallel with the investigation of the cause, uterine massage and oxytocic drugs are administered, followed by bimanual uterine compression, revision of the birth canal for laceration repair, if necessary, and uterine curettage. Crystalloid infusion and blood product transfusion are performed, if necessary. In case these measures are not sufficient, other resources should be rapidly implemented, such as uterine tamponade, transarterial embolization, ligation of uterine arteries, and B-Lynch suture. As last resort, hysterectomy should be performed. The more delayed the actions to control bleeding, the greater the blood loss and, consequently, the risk of coagulopathy, which may lead to hysterectomy, increased morbidity, hypovolemic shock, and maternal death.^2^

Regarding oxytocic drugs, several systematic reviews and double-blinded clinical trials indicated intravenous oxytocin as first-line treatment for PPH.^14 - 16^ Ergometrine also has an adequate effect on uterine contractility, but may cause secondary effects, such as nausea and vomiting, and is contraindicated for pregnant women with hypertension and heart disease (due to its alpha-adrenergic effect, with possible elevation of blood pressure), what makes it a second choice.^7 , 17^ Misoprostol comes as the third choice of treatment, when the use of oxytocin and methylergomevine has not ceased bleeding. If bleeding persists after the administration of misoprostol, surgical intervention is required.^2^

In places with scarce public health resources, misoprostol has several advantages as compared to oxytocin, since it is easy to administer, cheaper, thermostable, and has controllable adverse reactions, if given at low-doses.^10^ The use of misoprostol for treatment of PPH (800µg) may cause hyperpyrexia (1% to 14%), chills (37% to 47%), and fever (22% to 44%).^2 , 15 , 18 , 19^ These reactions depend on the dose administered, the optimal dose being 800µg.^7 , 12 , 14 , 18 , 19^ In this study, no adverse reactions were reported after the use of misoprostol, even in pregnant women who received a dose greater than 800µg.

Data in the literature indicate that approximately two thirds of pregnant women do not have a risk factor or past history that may be associated with the development of PPH.^20^ However, some predisposing factors to PPH have been mentioned, such as multiparity, conditions leading to uterine fatigue (prolonged, induced or accelerated delivery), amniotic fluid disorders (polyhydramnios and chorioamnionitis), uterine overdistension (fetal macrosomia and multiple pregnancy), premature labor, gestational anemia and coagulation disorders, preeclampsia, placenta abruptio and/or manual removal of the placenta, obesity, prolonged delivery associated with uterine infection, PPH in previous pregnancies, dystocia, women aged under 20 or above 35 years, inability to contract the uterine muscle due to the use of tocolytic agents, or general anesthesia. In such cases, greater vigilance during the pregnancy-puerperal cycle is necessary.^4 , 10 , 21 , 22^

Pregnant women with hypertensive syndromes, such as pregnancy-induced hypertension, hypertension and eclampsia, as well as those with infections that may lead to PPH, should also be under vigilance.^23^

Regarding the type of delivery, the chances of bleeding are lower in elective C-sections as compared to vaginal delivery and emergency cesarean sections.^24^ Regarding maternal mortality and the occurrence of severe bleeding, 96.3% of pregnant women presented at least one of the previously mentioned risk factors.^25^

In relation to the cause of PPH in this study, some women had secondary hemorrhage, which usually occurs due to uterine atony due to presence of placental fragments and/or infection, and the recommendation for treatment is similar to that of primary PPH (use of oxytocic drugs and in case of infection, antibiotics). For the others causes identified in the study, the recommendation is that the initial treatment should be for uterine atony (since it is the major cause of PPH), while reviewing the delivery canal for lacerations, placenta and its membranes to determine the cause and, if necessary, redirect management.^26^

All variables that may influence and predispose to PPH should be evaluated, allowing to predict and perform prophylactic interventions during pregnancy and delivery. In this context, prenatal visits and follow-up are crucial.^21^ The Ministry of Health recommends a minimum of six prenatal visits for each pregnant woman. The WHO, in turn, recommends eight consultations in order to reduce perinatal mortality and improve the contact of pregnant women with health services.^27 , 28^ In our study, most pregnant women had more than six prenatal visits, while 14% had less than five.

Prophylactic administration of an uterotonic drug soon after birth also helps to reduce the risk of PPH. The WHO and FIGO recommend the use of intravenous oxytocin for this purpose in all pregnant women, since it is possible to prevent at least half of the cases of PPH, and reduce its severity.^2 , 7 , 10^ Although there are recommendations for the prophylactic use of misoprostol, in the hospital of this study, there was no protocol established for this purpose, since there were other oxytocic drugs available (oxytocin and ergometrine) that had greater evidence to be used as first-line treatment.^2 , 29^

The success rate found with misoprostol to control PPH was relatively high (84.7%). Nevertheless, it should be noted that in many patients the drug was used only as third option, and its effects were added to those of the other oxytocic agents administered. In 13 cases it was used alone, and successful bleeding control was achieved in 92.3% of them. These results corroborate other studies, such as a cohort study conducted in three Nigerian hospitals, in which 800µg of sublingual misoprostol were administered to women with PPH caused by uterine atony and not exposed to other oxytocic agents during the third stage of labor. The study demonstrated 85% efficacy of misoprostol in stopping bleeding within 20 minutes of administration.^30^ A double-blinded non-inferiority trial of 978 women with primary PPH, not exposed to prophylactic oxytocic agents, showed a success rate of 90% with 800µg misoprostol (n=488), and of 96% with oxytocin (n=490) to treat hemorrhage.^15^

## CONCLUSION

The inclusion of misoprostol in the maternity hospital has offered an accessible and effective pharmaceutical option to control postpartum hemorrhage, including the cases in which the first procedures commonly adopted failed (oxytocin and ergometrine).

Despite the small number of cases investigated during the study period, the results obtained may reflect in quality of care provided to the pregnant women of the above-mentioned maternity hospital. Considering the recent provision of misoprostol in the organization, it is necessary to have uniform care actions to treat postpartum hemorrhage. Standardization and adoption of updated care protocols are therefore the way to follow.
