# Supplementary material for: Use of misoprostol in the treatment of postpartum hemorrhage: a pharmacoepidemiological approach
Source: Einstein (Sao Paulo). 2019 Oct 28;18:eAO5029. doi: 10.31744/einstein_journal/2020AO5029 (PMC6896658; doi:10.31744/einstein_journal/2020AO5029)
Supplement: Translation

# Translation: Uso do misoprostol no tratamento da hemorragia pós-parto: uma abordagem farmacoepidemiológica

**Authors:** Daeska Marcella Koch, Yanna Dantas Rattmann

**Affiliations:** 1 Universidade Federal do Paraná, Curitiba, PR, Brasil.

**Keywords:** Misoprostol, Parto obstétrico, Farmacoepidemiologia, Hemorragia pós-parto, Resultado do tratamento

## Abstract

**Objetivo:**

Caracterizar o uso do medicamento misoprostol para o tratamento da hemorragia pós-parto em gestantes.

**Métodos:**

Estudo observacional descritivo realizado por meio de dados secundários de gestantes que fizeram uso do misoprostol para tratamento da hemorragia pós-parto em maternidade pública de referência, no período de julho de 2015 a junho de 2017. Caracterizaram-se os perfis clínico e sociodemográfico das gestantes, o padrão de utilização do misoprostol e sua taxa de sucesso no controle da hemorragia pós-parto.

**Resultados:**

Foram identificadas 717 prescrições do misoprostol. Destas, 10% foram para tratamento da hemorragia pós-parto. Predominaram gestantes adultas jovens, casadas, com Ensino Médio completo, raça branca, da região urbana, multíparas (68,1%) e 25% apresentavam cesáreas prévias. A idade gestacional média foi 39 semanas e 51,4% das gestantes tiveram parto cesárea. Houve uso profilático de ocitocina em 47,2% das mulheres. O tratamento da hemorragia pós-parto eve sucesso em 84,7% das gestantes que usaram misoprostol. Destas, 79,2% também usaram ocitocina e 54,2% metilergometrina. Apenas 13,5% das gestantes tiveram menos de cinco consultas de pré-natal, e a principal causa da hemorragia pós-parto foi atonia uterina. Foram registrados 13 casos de complicações após a hemorragia, 15,3% necessitaram de hemotransfusão e houve um caso de óbito materno.

**Conclusão:**

O misoprostol demonstrou ser efetivo e seguro para o tratamento da hemorragia pós-parto.

## INTRODUÇÃO

Segundo a Organização Mundial da Saúde (OMS), a hemorragia pós-parto (HPP) é a causa de aproximadamente 25% de todas as mortes de gestantes no mundo, sobretudo em países de baixa renda, além de ser responsável por grande parte das morbidades maternas graves, como internações hospitalares prolongadas, necessidade de transfusões sanguíneas e procedimentos cirúrgicos que podem levar à perda da função reprodutiva.^( 1 )^No Brasil, as hemorragias constituem a segunda causa de morte materna, e a HPP contribui com 40,8% para o total das hemorragias obstétricas.^( 2 )^

Grande parte dessas mortes pode ser evitada pela profilaxia com ocitócitos durante a terceira fase do parto e do manejo rápido e adequado da hemorragia. Mesmo assim, acredita-se que, em média, 6% de todos os partos evoluam para a HPP, e 1,86% para a HPP grave (≥1.000mL de perda sanguínea).^( 3 )^

A principal causa de HPP é a atonia uterina (80% dos casos). Porém, esta condição também pode ser causada por lacerações do canal de parto ou períneo, inversão uterina, distúrbios de coagulação materna, retenção placentária, entre outras.^( 4 )^Caso venha a ocorrer HPP por atonia uterina, o tratamento inicial consiste em massagem uterina, seguida do uso de ocitócitos, como ocitocina, ergometrina, prostaglandinas e derivados.^( 2 )^

Dentre os fármacos ocitócitos, a primeira escolha para tratamento da HPP é a ocitocina intravenosa. Caso esta esteja indisponível ou se o sangramento persistir, é recomendado o uso da ergometrina, ou dose fixa de ocitocina e ergometrina combinadas. Como terceira opção, utiliza-se um fármaco de prostaglandina ou derivado, como o misoprostol.^( 2 , 5 )^

O misoprostol, devido à sua ação uterotônica, tem sido investigado como alternativa à ocitocina, pois é de fácil administração, estável à temperatura ambiente e apresenta baixo custo. Em 2006, a Federação Internacional de Ginecologia e Obstetrícia (FIGO) recomendou o uso do misoprostol para tratamento da HPP, principalmente em locais onde existam poucos recursos.^( 6 )^Em 2012, a FIGO publicou suas diretrizes indicando 800µg de misoprostol para o tratamento de HPP.^( 7 )^Contudo, foi somente em 2015 que a *World Health Organization* incluiu esse fármaco na lista de medicamentos essenciais para essa finalidade.^( 8 )^

O misoprostol é um ocitócito análogo da prostaglandina E1, originalmente desenvolvido para o tratamento de úlceras pépticas e duodenais. Na década de 90, o Cytotec^®^, seu nome comercial na época, adquirido nas drogarias ou no comércio ilegal, popularizou-se como método vastamente utilizado para induzir o aborto no Brasil. Em 1998, tornou-se medicamento sujeito a controle especial no país, com uso restrito a estabelecimentos hospitalares licenciados pela autoridade sanitária.^( 9 , 10 )^Atualmente, é considerado pela Federação Brasileira das Associações de Ginecologia e Obstetrícia (Febrasgo)^( 10 )^e pelo Ministério da Saúde^( 11 )^como alternativa farmacológica para maturação cervical e indução do parto com feto vivo; para interrupção da gravidez de feto anencéfalo, com risco para a mãe ou decorrente de estupro;^( 12 )^para abortamentos retidos e incompletos; e para tratamento da HPP.

Após sua administração (oral, sublingual, vaginal ou retal), o misoprostol sofre desesterificação no fígado, transformando-se no ácido misoprostol. Este metabólito ativo exerce ação direta nos receptores das prostaglandinas, provocando mudanças na estrutura físico-química do colágeno cervical, o que leva ao amolecimento e à maturação do colo uterino, favorecendo sua dilatação, além de promover aumento do cálcio intracelular, responsável pela contração do músculo miometrial. As reações adversas mais comuns associadas ao misoprostol são náuseas, vômitos, diarreias, dor abdominal, febre e calafrios. As reações graves são raras e consistem em taquisistolia, hipercontratilidade e ruptura uterina. Todas essas reações são dependentes da dose e tendem a reduzir em intensidade nas primeiras horas após a utilização.^( 10 - 13 )^

## OBJETIVO

Caracterizar o perfil clínico e epidemiológico do uso do medicamento misoprostol para tratamento da hemorragia pós-parto em gestantes.

## MÉTODOS

Trata-se de estudo observacional descritivo transversal, que visou estudar o padrão de utilização e a taxa de sucesso com o uso de o misoprostol para o tratamento da HPP, em todas as gestantes que fizeram uso deste medicamento em uma maternidade do estado do Paraná, Brasil. Este hospital integra a rede do Sistema Único de Saúde (SUS) e abriga um centro obstétrico de referência para gestação de alto risco para a cidade e a região metropolitana, onde ocorrem cerca de 3.000 partos ao ano.

O período compreendido pelo estudo foi de julho de 2015, quando a Vigilância Sanitária autorizou o uso do misoprostol no estabelecimento, até junho de 2017. Caracterizou-se ainda o perfil clínico dos recém-nascidos dessas gestantes, pela possibilidade de relação com a HPP.

Na análise da taxa de sucesso (efetividade) com o uso do misoprostol, foi considerado sucesso quando, após o uso do medicamento, a hemorragia cessou, sem necessidade de outros métodos complementares posteriores ou a ocorrência de complicações, como a histerectomia.

A OMS,^( 1 )^a FIGO^( 7 )^e o Ministério da Saúde^( 11 )^recomendam a quantidade de até 800µg do misoprostol para o tratamento da HPP, administrada em até quatro comprimidos consecutivos de 200µg. Embora os comprimidos de misoprostol sejam desenvolvidos para administração vaginal, as vias de administração preferenciais para a HPP são a sublingual ou a retal. O hospital adotava a via retal em sua rotina.

Os dados foram coletados por meio do formulário de solicitação de misoprostol, nos quais foram obtidas informações sobre idade materna, idade gestacional (calculada a partir da ultrassonografia, em semanas completas), justificativa de uso do medicamento (por meio da Classificação Estatística Internacional de Doenças e Problemas Relacionados com Saúde – CID-10) e a posologia; das prescrições médicas, com posologia, tempo de tratamento e uso de outros medicamentos ocitócitos (metilergometrina e/ou ocitocina); do sistema informatizado (cor/raça, escolaridade, estado civil e local de residência); e do prontuário eletrônico, no qual constavam tipo parto realizado, justificativa para realização de cesárea, sucesso com o uso do medicamento, gestações anteriores, paridade, complicações no parto ou pós-parto, condições existentes durante a gestação (comorbidades materna e condições no feto), número de consultas de pré-natal e outros métodos auxiliares utilizados para a realização do parto. Dados do concepto (Apgar no primeiro e no quinto minuto de vida, e peso ao nascer) também foram coletados para análise.

Os dados coletados foram armazenados no Microsoft Excel^®^, com posterior análise descritiva pelo aplicativo Epi Info (versão 6.04).

O estudo obteve aprovação do Comitê de Ética em Pesquisa da Universidade Federal do Paraná, Setor de Ciências da Saúde, conforme parecer 017569/2017, CAAE: 65363617.2.0000.0102.

## RESULTADOS

O misoprostol foi administrado a 717 gestantes, das quais 362 receberam o medicamento para indução do trabalho de parto com o feto vivo (50,5%), 283 para manejo de aborto e interrupção da gestação com feto morto e retido (39,5%), e 72 para tratamento da HPP (10,0%).

As características sociodemográficas e as informações clínicas das gestantes que utilizaram o misoprostol para tratamento da HPP são apresentadas nas tabelas 1 e 2 , respectivamente.

**Tabela 1:** Variáveis sociodemográficas das gestantes tratadas com misoprostol para hemorragia pós-parto

Variáveis	
Faixa etária (anos)	
<20	10 (13,9)
20-34	45 (62,5)
>34	17 (23,6)
Estado civil	
Solteira	15 (20,8)
Casada/união estável	47 (65,3)
Viúva/divorciada	0 (0)
Não informado	10 (13,9)
Escolaridade	
Alfabetizada	1 (1,4)
Fundamental Incompleto	9 (12,5)
Fundamental Completo	10 (13,9)
Médio Incompleto	5 (6,9)
Médio Completo	12 (16,7)
Superior Incompleto	5 (6,9)
Superior Completo	1 (1,4)
Não informado	29 (40,3)
Raça/cor	
Branca	37 (51,4)
Parda	31 (43,0)
Preta	1 (1,4)
Amarela	1 (1,4)
Não informado	2 (2,8)
Local de residência	
Urbana	52 (72,2)
Rural	20 (27,8)

Resultados expressos por n (%).

**Tabela 2:** Variáveis clínicas das gestantes tratadas com misoprostol para hemorragia pós-parto

Variáveis	
Idade gestacional, semanas	
<12	1 (1,4)
12-36	10 (13,9)
37-41	61 (84,7)
Paridade	
0	23 (31,9)
1	23 (31,9)
2	11 (15,3)
3	5 (6,9)
4	5 (6,9)
5	3 (4,2)
6	2 (2,8)
Gemelaridade	3 (4,2)
Antecedentes de parto vaginal	32 (44,4)
Antecedentes de aborto	16 (22,2)
Antecedentes de cesáreas	18 (25,0)

Resultados expressos por n (%).

A média de idade foi 28,51±7,46 anos, variação de 16 a 45 anos, e mediana da idade gestacional de 39 semanas, variando de 7 a 41 semanas.

A taxa de sucesso do uso de misoprostol para o tratamento da HPP, associado ou não a outros medicamentos, foi 84,7%, sendo utilizado como terceira opção em 37 casos (51,4%), como segunda opção em 22 casos (30,5%), e utilizado isoladamente em 13 casos (18,0%). Das gestantes que utilizaram misoprostol como única terapia, somente uma não teve sucesso, necessitando de intervenção cirúrgica e histerectomia ( Tabela 3 ).

**Tabela 3:** Variáveis relacionadas ao parto e ao uso de medicamentos das gestantes tratsadas com misoprostol para hemorragia pós-parto

Variáveis	
Parto	
Vaginal	34 (47,2)
Cesárea	37 (51,4)
Outros (curetagem)	1 (1,4)
Uso profilático de ocitocina	34 (47,2)
Uso de ocitocina após HPP	57 (79,2)
Uso de metilergometrina após HPP	39 (54,2)
Quantidade de comprimidos misoprostol 200µg	
1	3 (4,2)
2	10 (13,9)
3	1 (1,4)
4	56 (77,8)
5	1 (1,4)
6	1 (1,4)
Taxa de sucesso após o uso do misoprostol	61 (84,7)

Resultados expressos por n (%). HPP: hemorragia pós-parto.

Dos 11 casos de HPP que não tiveram sucesso no manejo, todos utilizaram ocitocina como primeira opção, cinco utilizaram metilergometrina, e todos usaram misoprostol, variando nas posologias de um (n=1), dois (n=1), três (n=1) e quatro comprimidos de 200µg (n=8).

Observou-se que 54% das gestantes fizeram uso de metilergometrina, e, destas, quatro tinham hipertensão, fato contraindicado para o uso deste medicamento.

Conforme prontuários médicos, seis casos de HPP ocorreram devido à laceração do canal de parto (8,3%), um caso devido à perfuração da parede uterina (no procedimento de curetagem; 1,4%), dois casos eram de HPP secundária (que ocorre entre 24 horas e 6 semanas após o parto, devido a restos placentários e infecção uterina; 2,8%), três casos se deram pelo distúrbio de coagulação (4,2%) e os demais (60 casos; 83,3%) devido à atonia uterina. Um total de 11 (15,3%) gestantes necessitou de hemotransfusão após a HPP.

Das 72 gestantes atendidas para tratamento da HPP, 51,4% tiveram o parto cesárea, sendo as principais justificativas para a ocorrência desse parto a desproporção cefalopélvica (13,5%), a falha da indução do parto vaginal (13,5%) e a macrossomia fetal (8,1%).

Foram referidos 24 diferentes CIDs para a utilização do medicamento. Dentre eles, aproximadamente 28% correspondeu a O721 (outras hemorragias do pós-parto imediato), 15,3% a O72 (HPP) e 9,7% a O800 (parto espontâneo cefálico).

Sobre o número de consultas de pré-natal, 48,6% das gestantes compareceram entre seis e dez consultas, 22,2% compareceram a mais de dez consultas, 15,3% não tinham dados arrolados, e 13,9% compareceram entre duas a cinco consultas.

Relativo aos dados dos recém-nascidos, foram identificados 5,3% de óbitos fetais, 12,0% com macrossomia (peso ≥4.000 g) e 8,0% com baixo peso (<2.500 g). Do total, 82,7% nasceram com Apgar acima de 7 no primeiro minuto após o nascimento, e 89,3% com Apgar acima de 7 no quinto minuto de vida.

Foram identificadas 13 complicações maternas após o parto e tratamento da hemorragia, dentre elas histerectomia (n=4), choque hipovolêmico com posterior internamento em unidade de terapia intensiva (n=4), anemia (n=2), infecção após o parto (n=2) e óbito materno (n=1).

Dentre as condições preexistentes e gestacionais, foram encontradas 121 ocorrências nas 72 gestantes, conforme apresentado na tabela 4 , sendo as mais prevalentes a doença hipertensiva específica da gestação (DHEG), com 12,4%, seguida do hipotireoidismo e da obesidade, com 9,1% cada.

**Tabela 4:** Condições preexistentes e gestacionais das gestantes tratadas com misoprostol para hemorragia pós-parto (n=121)

Condições existentes	
Doença hipertensiva específica da gestação	15 (12,4)
Hipotiroidismo	11 (9,1)
Obesidade	11 (9,1)
Infecção do trato urinário	10 (8,3)
Parto prematuro	10 (8,3)
Hipertensão	7 (5,8)
Infecção vaginal	7 (5,8)
Distúrbios neurológicos*	6 (5,0)
Tabagismo	5 (4,1)
Presença de edema	4 (3,3)
*Diabetes mellitus*	3 (2,5)
Doença do trato respiratório	3 (2,5)
Gemelaridade	3 (2,5)
Infecção intestinal	3 (2,5)
Plaquetopenia	3 (2,5)
Pré-eclâmpsia	3 (2,5)
Anemia	2 (1,7)
Distúrbios do líquido amniótico	2 (1,7)
Sífilis	2 (1,7)
Varizes uterinas	2 (1,7)
Bartolinite	1 (0,8)
Corioamnionite	1 (0,8)
Descolamento de placenta	1 (0,8)
Ex-tabagista	1 (0,8)
Eclâmpsia	1 (0,8)
Hérnia umbilical	1 (0,8)
Hiperêmese gravídica	1 (0,8)
Sangramento vaginal	1 (0,8)
Útero bicorno	1 (0,8)
Total	121 (100,0)

Resultados expressos por n (%). * Síndrome do pânico, depressão, ansiedade, esquizofrenia e/ou epilepsia.

Neste estudo, todas as gestantes apresentaram ao menos um fator de risco que poderia culminar na HPP, como idade menor que 20 anos e maior que 35 anos (37,5%); multiparidade (68,1%), gemelaridade (4,2%), parto induzido (1,4%), distúrbios do líquido amniótico (2,8%), macrossomia fetal (12%), trabalho de parto prematuro (13,9%), anemia gestacional ou plaquetopenia (6,9%), distúrbios hipertensivos (36,1%), corioamnionite (1,4%), descolamento de placenta (1,4%), obesidade (15,3%), parto associado à infecção uterina (1,4%), distócias de progressão (2,8%), além da realização de parto normal em 47,2% das gestantes.

## DISCUSSÃO

A hemorragia é uma das principais causas evitáveis de morte materna.^( 1 )^Quando diagnosticada uma HPP, paralelamente à investigação da causa, procede-se à realização da massagem uterina e à administração de ocitócitos, seguidas de compressão uterina bimanual, revisão do canal de parto para reparo de lacerações, se necessário, e curetagem uterina. Se necessário, são feitas a infusão de cristaloides e a transfusão de hemoderivados. Caso essas medidas não sejam suficientes, devem ser realizados outros métodos rapidamente, como tamponamento uterino, embolização transarterial, ligadura de artérias uterinas e sutura de B-Lynch. Como último recurso, deve ser realizada a histerectomia. Quanto mais postergadas as ações para controle da hemorragia, maior a perda sanguínea e, consequentemente, o risco de coagulopatia, podendo levar a histerectomia, aumento da morbidade, choque hipovolêmico e óbito materno.^( 2 )^

Em relação aos medicamentos ocitócitos, diversas revisões sistemáticas e ensaios clínicos duplo-cegos indicam a ocitocina intravenosa como primeira linha para tratamento da HPP.^( 14 - 16 )^A ergometrina também possui efeito adequado na contratilidade uterina, mas apresenta efeitos secundários, como náuseas e vômitos, sendo contraindicada para gestantes hipertensas e com cardiopatias (devido a seu efeito alfa-adrenérgico, com possível elevação da pressão arterial), o que a torna segunda escolha.^( 7 , 17 )^Nesse sentido, o misoprostol atua como terceira escolha de tratamento, quando o uso de ocitocina e de metilergometrina não tiverem cessado o sangramento. Caso a hemorragia persista após administração do misoprostol, é necessária a intervenção cirúrgica.^( 2 )^

Em locais com poucos recursos de saúde pública, o misoprostol apresenta diversas vantagens, quando comparado à ocitocina, pois é de fácil administração, tem menor custo, é termoestável e apresenta reações adversas contornáveis a baixas doses.^( 10 )^O uso de misoprostol para tratamento de HPP (na quantidade de 800µg) pode causar hiperpirexia (1% a 14%), calafrios (37% a 47%) e febre (22% a 44%).^( 2 , 15 , 18 , 19 )^Estas reações dependem da dose administrada, sendo a dose ótima 800µg.^( 7 , 12 , 14 , 18 , 19 )^No presente estudo, não foram relatadas reações adversas após o uso de misoprostol, mesmo naquelas gestantes que ultrapassaram 800µg.

Dados da literatura indicam que aproximadamente dois terços das gestantes não apresentam fator de risco ou histórico que possa estar associado ao desenvolvimento da HPP.^( 20 )^Porém, são citados alguns fatores que predispõem à HPP, para os quais há a necessidade de maior vigilância durante o ciclo gravídico-puerperal, como multiparidade, condições que levam à fadiga uterina (parto prolongado, induzido ou acelerado), distúrbios do líquido amniótico (polidrâmnia e corioamnionite), situações de hiperdistensão uterina (macrossomia fetal e gemelaridade), trabalho de parto prematuro, anemia gestacional e distúrbios de coagulação, pré-eclâmpsia, descolamento prematuro e/ou retirada manual da placenta, obesidade, parto prolongado associado à infecção uterina, HPP em gestações anteriores, distócias, mulheres com idade menor que 20 anos e acima de 35 anos ou com inabilidade de contrair o músculo uterino devido ao uso de tocolíticos ou anestesia geral.^( 4 , 10 , 21 , 22 )^

Também devem ser consideradas as gestantes com síndrome hipertensiva, como DHEG, hipertensão e eclâmpsia, além daquelas com presença de infecções que podem culminar em HPP.^( 23 )^

Em relação ao tipo de parto, as chances de hemorragia são menores nos partos finalizados em cesariana eletivas comparadas aos partos vaginais e às cesáreas de emergência.^( 24 )^Em relação à mortalidade materna e à ocorrência de hemorragia grave, 96,3% das gestantes apresentam pelo menos um dos fatores de risco citados previamente.^( 25 )^

Quanto à causa da HPP no presente estudo, algumas mulheres apresentaram hemorragia secundária, que ocorre normalmente devido à atonia uterina secundária à presença de fragmentos placentários e/ou infecção, sendo a recomendação de tratamento semelhante ao da HPP primária (uso de ocitócitos e, no caso de infecção, de antibióticos). Para as demais causas identificadas no estudo, a recomendação é que o tratamento inicial deve ser baseado na atonia uterina (já que é a principal causa de HPP), enquanto procede-se à revisão do trajeto do parto, em busca de lacerações e da placenta e suas membranas, a fim de determinar a causa e, se necessário, redirecionar para o correto tratamento da hemorragia.^( 26 )^

Todas as variáveis que podem influenciar e predispor à HPP devem ser avaliadas, permitindo prever e realizar intervenções profiláticas durante a gestação e a assistência ao parto. Nesse contexto, as consultas e o acompanhamento do pré-natal têm valor fundamental.^( 21 )^O Ministério da Saúde preconiza o número mínimo de seis consultas de pré-natal para cada gestante. A *World Health Organization,* por sua vez, recomenda oito consultas para que haja redução da mortalidade perinatal e melhore o contato da gestante com os serviços de saúde.^( 27 , 28 )^No presente estudo, a maioria das gestantes teve mais do que seis consultas de pré-natal, enquanto aproximadamente 14% tiveram menos do que cinco consultas.

A administração profilática de um uterotônico logo após o nascimento do bebê também auxilia na redução do risco de HPP. A OMS e a FIGO recomendam o uso de ocitocina intravenosa para essa finalidade em todas as gestantes, a qual é capaz de evitar no mínimo metade dos casos de HPP, além de reduzir sua gravidade.^( 2 , 7 , 10 )^Apesar de haver recomendações do uso de misoprostol para profilaxia, no hospital em estudo não havia protocolo de uso estabelecido para esta finalidade, uma vez que existiam outros ocitócitos disponíveis (ocitocina e ergometrina) e com maiores evidências para serem usados como primeira linha de tratamento.^( 2 , 29 )^

A taxa de sucesso encontrada com o uso de misoprostol para controle da HPP foi relativamente alta (84,7%). Porém, deve-se observar que, em muitas pacientes, o medicamento foi utilizado somente como terceira opção, tendo seus efeitos sido somados aos dos outros ocitócitos administrados. Nos 13 casos em que foi utilizado isoladamente, teve taxa de sucesso de 92,3% no controle da hemorragia. Esses resultados corroboram outros estudos, como um de coorte realizado em três hospitais da Nigéria, no qual foram administrados 800µg de misoprostol sublingual em mulheres com HPP causada por atonia uterina e não expostas a outros ocitócitos durante o terceiro estágio do trabalho de parto. O estudo demostrou eficácia do misoprostol de 85% ao cessar o sangramento em 20 minutos após sua administração.^( 30 )^Um ensaio duplo-cego de não inferioridade realizado com 978 mulheres não expostas a ocitócitos profiláticos e com HPP primária mostrou taxa de sucesso de 90% com o uso de 800µg de misoprostol (n=488) e de 96% com o uso de ocitocina (n=490) para tratamento da hemorragia.^( 15 )^

## CONCLUSÃO

A inclusão do misoprostol no hospital maternidade ofereceu opção farmacológica acessível e efetiva para o controle da hemorragia pós-parto, inclusive nos casos de falha dos primeiros procedimentos comumente adotados (administração de ocitocina e ergometrina).

Apesar do reduzido número de casos investigados no período deste estudo, os resultados obtidos podem refletir na qualidade do atendimento e das ações assistenciais prestadas às gestantes na referida maternidade. Considerando a oferta recente do misoprostol no estabelecimento, percebe-se a necessidade de uniformização das ações médico-assistenciais no tratamento da hemorragia pós-parto. Neste contexto, a padronização e a aderência a protocolos assistenciais atualizados são um caminho a ser seguido.
